# Effects of health-related dispositions on citizens’ appraisals toward the COVID-19 pandemic and protective behavior

**DOI:** 10.1371/journal.pone.0305995

**Published:** 2024-09-05

**Authors:** Xinyuan Fu, Ruoran Fu, Shuxian Li, Xiaona Du, Mei Zhang, Jiaxin Duan, Hanmin Wang, Guixin Li

**Affiliations:** 1 Department of Psychology, School of Sociology and Psychology, Central University of Finance and Economics, Beijing, China; 2 Faculty of Health, School of Psychology & Counselling, Queensland University of Technology, Brisbane, Australia; 3 Faculty of Education, Department of Educational Psychology and Counselling, University of Malaya, Kuala Lumpur, Malaysia; University of Hong Kong Faculty of Social Sciences, HONG KONG

## Abstract

In this study, health risk attitude and health locus of control were included as dispositional factors in the Protection Motivation Theory (PMT) to explain people’s protective behavior in the context of COVID-19 pandemic. Empirical data involved two waves of data with a sample of 526 adults with full-time jobs from Beijing, China, and structural equation model results confirmed a partial successful extension of the PMT. Specifically, health risk attitude had a direct effect on citizens’ protective behavior, but without an indirect effect mediated by threat appraisal toward the COVID-19 pandemic; health locus of control did not directly associate with citizens’ protective behavior, but had an indirect effect on it fully via coping appraisal toward the COVID-19 pandemic. Thus, the PMT has been extended by adding a distal dispositional factor on the impact of coping appraisal on protective behavior. Implications for advancing the government’s anti-epidemic strategy are discussed.

## 1. Introduction

Human beings have plenty of experiences in responding to public health emergencies, such as the SARS and Ebola, yet are unable to adopt protective behaviors timely and effectively, such as failing to wear masks and keep physical distance during the COVID-19 pandemic. To explore what stops people from adopting protective behaviors, scholars have conducted a lot of research and used different theories. Among the theories used in explaining protective behavior, Protection Motivation Theory (PMT; [1]) has displayed its powerful explanation across different healthy and protective behaviors, such as the preventive behavior [2] and vaccination during COVID-19 [3], owing to its key components threat appraisal and coping appraisal. However, much empirical evidence indicates that people could not evaluate the threat and their own coping efficacy appropriately [4,5]. Also, governments and social organizations around the world tried to boost citizens’ threat and coping appraisals via various measures, however, with limited success [6]. These failed attempts hint the pivot of invalidity of the PMT, which might be engendered by the diverse factors driving threat and coping appraisals, including not only environmental factors but also dispositional factors. Therefore, the present study was designed to extend the PMT by exploring the effects of health-related dispositions on individuals’ appraisals and thus their protective behaviors, piloting on a sample of adults in Beijing, China in the COVID-19 pandemic.

According to the PMT, people protect themselves based on two factors: threat appraisal and coping appraisal [1]. Basically, these two factors have formed a closed loop drawing the cognitive appraisal when people confront external threats from maladaptive and adaptive perspectives individually [7]. Threat appraisal is maladaptive assessment which consists of the perceived severity of a threatening event and the perceived vulnerability of the occurrence, whereas coping appraisal is an adaptive evaluation which consists of perceived response efficacy and perceived self-efficacy [8,9]. Citizens’ threat and coping appraisals toward the COVID-19 pandemic are largely determined by situational factors (e.g., the severity of the situation; [10]), yet whether they are impacted by dispositional factors is relatively neglected. In this regard, some empirical evidence points out directly that health risk attitude would affect people’s threat appraisal [11,12], and health locus of control is one of the factors influencing individuals’ self-efficacy, a manifestation of coping appraisal [13]. More importantly, health risk attitude and threat appraisal, and health locus of control and coping appraisal have similar mental content, but they are on different mental levels [14,15]. Health risk attitude and health locus of control are located on general dispositional levels, while threat appraisal and coping appraisal are on context-specific state levels [15]. Therefore, we focused on health risk attitude and health locus of control in the present study, which may impact threat appraisal and coping appraisal respectively, and then impact protective behavior of citizens.

### 1.1 Health risk attitude, threat appraisal, and protective behavior

Health risk attitude refers to individual’s utility function derived from a series of risky choices that pose a threat to health [16]. It reflects a person’s standing on the continuum from risk seeking to risk aversion, and is commonly considered to be a personality trait [17,18]. Theoretically and empirically, health risk attitude has been found to link to one’s protective behavior [19]. For instance, people with higher levels of health risk aversion are less likely to engage in unhealthy behaviors such as smoking and drug abuse [20,21], and are more likely to conduct self-preservation behaviors such as wearieeng masks and social distancing in the pandemic of COVID-19 [22]. Individuals with different health risk attitudes respond differently to fear appeals; typically, individuals with health risk preference have relatively low levels of fear arousal, whereas those with health risk aversion have relatively high levels of fear arousal [23]. According to the PMT, protection motivation and behavior originate largely from individual’s fear, which can influence one’s perceived threat and thus initiate subsequent behavioral process [7,12]. We further believe that threat/fear appraisal is influenced by one’s health risk attitude. In other words, threat appraisal may be a potential mediator between health risk attitude and citizens’ protective behavior. Thus, it is reasonable to speculate that health risk attitude (i.e., the continuum from risk preference to risk aversion) would positively predict threat appraisal, which would positively predict protective behavior of citizens. In this regard, this research aims to investigate the relationship between health risk attitude and protective behavior, and whether this link would be mediated by threat appraisal toward the COVID-19 pandemic.

### 1.2 Health locus of control, coping appraisal, and protective behavior

Health locus of control is defined as the degree to which people believe that they, as opposed to external forces (beyond their influence), have control over the outcome of events concerning their health [24]. It reflects one’s locus of control in the health domain and is a personality variable linked with generalized expectancies about the future [25]. Individuals with a strong internal health locus of control believe events concerning their health are primarily a result of their own actions; people with an external health locus of control tend to believe that the things which happen regarding their health are out of their control, and believe health related events are a result of external factors, such as fate, luck, and the influence of powerful others like doctors, the police, or government officials [26]. Empirical data on health locus of control in a number of fields has shown that internal health locus of control is related to various health-related behaviors, such as weight control, smoking cessation, and taking daily medication to control blood pressure [27,28].

More recently, internal health locus of control has been found to relate indirectly and positively to citizens’ protective behavior against the COVID-19 pandemic [29]. Moreover, health locus of control is linked to individual’s coping appraisal (i.e., perceived response efficacy and perceived self-efficacy) toward health threats [13]. Likewise, based on the PMT, we believe people who have internal health locus of control have higher expectation that carrying out recommended action will remove health threat and have stronger belief in own ability to execute recommended action successfully [30,31]. On this matter, it is logical to assume that coping appraisal may be a potential mediating variable between health locus of control and protective behavior; internal health locus of control would positively predict coping appraisal, which would positively predict protective behavior. Therefore, this research also investigates the relationship between health locus of control and protective behavior, and whether this link could be mediated by coping appraisal toward the COVID-19 pandemic.

### 1.3 The current study

**Fig 1:**
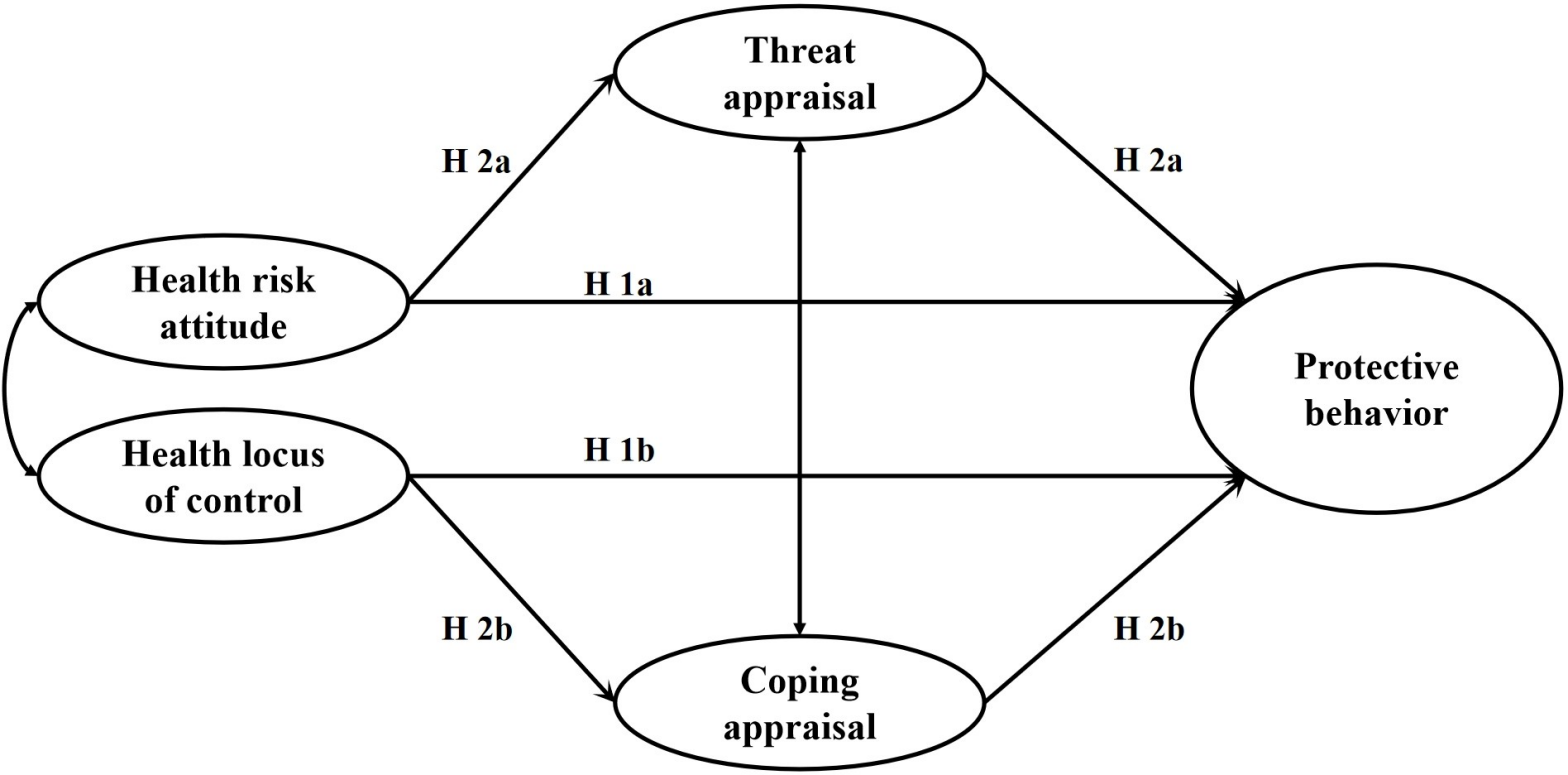
Theoretical model for the effects of health-related dispositions on citizens’ appraisals toward the COVID-19 pandemic and protective behavior.

This study examines the effects of health-related dispositions on citizens’ appraisals toward the COVID-19 pandemic and protective behavior via a two-time-point data collection strategy. Based on the PMT, we specifically explore the effects of health risk attitude and health locus of control on threat appraisal and coping appraisal toward the COVID-19 pandemic and protective behavior of citizens. We also investigate whether threat appraisal and coping appraisal toward the COVID-19 pandemic would respectively mediate the effects of health risk attitude and health locus of control on protective behavior. Our theoretical model is displayed in Fig 1. We hypothesized that health risk attitude (from risk preference to risk aversion) would positively predict protective behavior (Hypothesis 1a); internal health locus of control would positively predict protective behavior (Hypothesis 1b). We further hypothesized that health risk attitude would positively predict threat appraisal toward the COVID-19 pandemic, which in turn would positively predict protective behavior (Hypothesis 2a); internal health locus of control would positively predict coping appraisal toward the COVID-19 pandemic, which in turn would positively predict protective behavior (Hypothesis 2b).

It is noteworthy that we do not consider any cross-cutting paths owing to emphasis on the parallel process of threat appraisal and coping appraisal in the PMT, which does not include the interacting effect between threat appraisal and coping appraisal [2,3]. The independence of definitions between the two sets of variables (i.e., health risk attitude and threat appraisal; health locus of control and coping appraisal) also drives the current study not to consider the cross-cutting paths. Moreover, gender, age, and education level have been found to have great impacts on individuals’ protective behaviors during the COVID-19 pandemic, as well as residence and subjective distance from the epidemic [10,32]. Thus, when testing the hypotheses, participant’s gender, age, education level, residence (i.e., urban, suburban, and rural), and subjective distance from the epidemic were controlled. Data files, syntax files, and log files for the analyses are in S1 File.

## 2. Methods

### 2.1 Participants

In total, 558 adults with full-time jobs were recruited at Time 1 between December 30^th^ and 31^st^, 2020, and 526 of them passed the attention check (337 females, 189 males; *M*_age_ = 28.23, *SD*_age_ = 7.94, range _age_ = 18–66 years). Approximately one month later, 431 of them participated at Time 2 between January 29^th^ and February 1^st^, 2021, and 402 participants passed the attention check (257 females, 145 males; *M*_age_ = 28.58, *SD*_age_ = 7.89, range _age_ = 18–66 years). Attention checks included “please choose ‘disagree’ for this item” at Time 1 and “please choose ‘strongly agree’ for this item” at Time 2, respectively, for data quality control. In the initial sample (*n* = 526), 0.38% of participants had a middle school diploma or lower, 5.13% of participants possessed a high school diploma or similar education level, 8.37% of participants had a college degree, 51.90% of participants were bachelor’s degree holders, and 34.22% of participants had a master’s degree or higher. Among the initial valid 526 participants, 451 of them were urban citizens, 56 were suburban citizens, and 19 were rural citizens. Of the 402 valid participants who participated at both Time 1 and 2, 323 were urban citizens, 46 were suburban citizens, and 33 were rural citizens. The sample was recruited from Beijing, China. It needs to be clarified that because the adults with full-time jobs typically have relatively high spatial mobility and engage in frequent social interactions to earn a living, it is crucial to understand their attitudes and behaviors toward the COVID-19 pandemic. Therefore, we recruited adults with full-time jobs in the current study.

Following this logic, detailed contextual information about the COVID-19 pandemic during the data collection period also needs to be clarified. After nearly six months without an outbreak, on December 23^rd^, 2020, one asymptomatic case of COVID-19 was reported in Beijing, and a total of 16 cases were involved as of December 29^th^, 2020. As of January 1^st^, 2021, Beijing reported a total of 25 cases, signaling the first peak of the outbreak during which we collected the first wave of data. From January 1^st^ to 18^th^, 2021, the outbreak was under initial control, with the number of new cases ranging from 0 to 2 per day. However, on January 19^th^, Beijing hit the second peak with 7 new cases. With urgent control measures, from January 20^th^ to January 26^th^, Beijing reported a daily increase of 2 to 4 cases. From January 27^th^ to February 1^st^, there were 0 to 2 new cases daily, signaling a downward trend in the outbreak during which we collected the second wave of data. After 5 new cases on February 3^rd^ and 2 new cases on February 6^th^ were reported, the outbreak ended completely on February 7^th^, 2021. Accordingly, our two waves of data were collected at the time when citizens were responding to the COVID-19 pandemic.

### 2.2 Procedure

In conducting the research reported in this article, we have complied with APA ethical standards in the treatment of human subjects. The procedure was approved by Institutional Review Board (IRB) of Central University of Finance and Economics. Convenience sampling was used. The participants were recruited and online-tested through the Credamo (www.credamo.com). Credamo is a professional integrated data platform, providing services including questionnaire design, millions of online subjects, data collection, visual statistical modeling, and other functions for research and business application. We posted our questionnaire and the requirements for participants on the website. We called for Beijing citizens who were eighteen years of age or older to take our anonymous and paid survey about their behaviors in response to the COVID-19 pandemic. Eligible subjects could click the questionnaire link and complete it. At each time point, the survey took approximately three minutes. All the participants were informed of the survey data confidentiality and signed the written consent form online. They were debriefed and paid online at both junctures of the study. Health risk attitude, health locus of control, and the demographic factors (i.e., gender, age, education level, and residence) were measured at Time 1. Threat appraisal and coping appraisal toward the COVID-19 pandemic, protective behavior, and subjective distance from the epidemic were measured at Time 2. With this two-time-point data collection strategy, common method bias could be reduced.

As the measures were derived from English, we hired two English major graduates (whose mother tongue is Chinese) to translate them into Chinese using Brislin translation model to ensure accuracy [33]. The specific steps were as follows. Firstly, one of them independently translated the original scales into Chinese; then, the other one independently translated the Chinese version of the scales back into English; finally, two of them compared and discussed the original scales, the Chinese ones, and the back-translated ones together, and determined the final Chinese version of the scales. Results of confirmatory factor analysis showed that the Chinese version of the scales had good validity. Factor loadings for all the measures are shown in S1 Table.

### 2.3 Measures

#### 2.3.1 Health risk attitude

Health risk attitude was assessed using nine items taken from the health/safety risk perception subscale within the Domain-Specific Risk-Attitude Scale [16]. Participants were informed that “people are often in risky situations that contain uncertainty about what the consequences will be and there is the possibility of bad consequences. However, riskiness is a very personal and intuitive notion. We are interested in your gut-level assessment of how risky each of the following situations is.” Then they were asked to evaluate how risky the action or behavior for him/her was in each item using a 5-point Likert scale, ranging from 1 (not at all risky) to 5 (extremely risky). Sample items included “ignoring some persistent physical pain by not going to the doctor”, “never using sunscreen when you sunbathe” and “eating ‘expired’ food products that still ‘look okay’”. Notably, the risky situation in each item happens commonly in life, and the riskiness highly depends on personal and subjective evaluation rather than general recognition of the situation [16]. Thus, assessment of riskiness via this measure could indicate one’s health risk preference or aversion. Higher mean scores indicated higher levels of health risk aversion, and lower mean scores indicated higher levels of health risk preference. The value for McDonald’s omega was .77 in the study sample. A latent variable for health risk attitude was created, and the model fit of confirmatory factor analysis with modification was acceptable, χ^2^ (26) = 74.69, *p* < .001, CFI = .969, TLI = .957, RMSEA = .060, SRMR = .036. Factor loadings ranged from .44 to .66.

#### 2.3.2 Health locus of control

Health locus of control was measured using five items taken from the internal subscale of the Health Locus of Control Scale [24]. The participants rated each item on a 6-point Likert scale ranging from 1 (strongly disagree) to 6 (strongly agree). Sample items included “if I take care of myself, I can avoid illness” and “I am directly responsible for my health”. Higher mean values indicated higher levels of internal health locus of control (McDonald’s omega = .65). A latent variable was created, and the model fit for confirmatory factor analysis with modification was acceptable, χ^2^ (3) = 4.62, *p* = .202, CFI = .997, TLI = .991, RMSEA = .032, SRMR = .011. Factor loadings ranged from .46 to .64.

#### 2.3.3 Threat appraisal

Threat appraisal toward the COVID-19 pandemic was measured using five items modified from Kim et al. [34] and Zhang et al. [10]. The items were “I think I will probably get infected with COVID-19”, “I think the COVID-19 epidemic in China is serious”, “I feel close to the COVID-19 pandemic”, “I worry about getting infected with COVID-19”, and “I feel vulnerable to the COVID-19 infection”. The participants responded on a 5-point Likert scale ranging from 1 (very much unlike me) to 5 (very much like me). Higher average scores suggested that the participants appraised the virus more as a threat (McDonald’s omega = .79). A latent variable was created, and the model fit for confirmatory factor analysis with modification was acceptable, χ^2^ (1) = 1.23, *p* = .268, CFI = 1.000, TLI = .999, RMSEA = .024, SRMR = .004. Factor loadings ranged from .43 to .84.

#### 2.3.4 Coping appraisal

Coping appraisal toward the COVID-19 pandemic was measured using two items adapted from Kim et al. [34]. The items were “I feel confident that I can protect myself from the COVID-19 pandemic” and “I have little control over whether I contract COVID-19 or not (reverse coded)”. The participants responded on a 7-point Likert scale ranging from 1 (strongly disagree) to 7 (strongly agree). Higher means suggested the participants had higher expectation that carrying out proper action will remove the COVID-19 threat and had stronger belief in own ability to execute proper action successfully (*r* = .31, *p* < .001).

#### 2.3.5 Protective behavior

Protective behavior was assessed using eight items developed based on the advice from WHO on ways to protect oneself and prevent the spread of COVID-19 [35]. Sample items included “keep physical distance of at least 1 meter from others” and “thoroughly clean hands with either an alcohol-based hand rub or soap and water”. Participants were asked to rate how often they had done the protective behaviors in the past week on a 5-point Likert scale ranging from 1 (never) to 5 (always). Notably, if the circumstance in the item did not apply to participants, they could choose the option “not applicable”. This answer was coded as missing due to item inapplicability. Nine items were created initially, but the results of exploratory factor analysis showed that one item (i.e., cover mouth and nose with a bent elbow or tissue when coughing or sneezing) formed a separate component. This item was more about protecting others than protecting self, and hence was removed. Then the results of both exploratory factor analysis and confirmatory factor analysis with modification showed that, the final eight items formed a single factor, χ^2^ (15) = 34.08, *p* = .003, CFI = .990, TLI = .981, RMSEA = .056, SRMR = .027. Factor loadings ranged from .49 to .75. The value for McDonald’s omega was .84 in the study sample.

#### 2.3.6 Subjective distance from the epidemic

Subjective distance from the epidemic was measured via a single question “to the best of your knowledge, of those infected with the new coronavirus, where do the nearest people to you live?” The participants answered it by selecting one of the following four options: “in the same neighborhood as me (coded as 1)”, “in a nearby neighborhood (coded as 2)”, “in the same district as me (coded as 3)”, and “in the same city as me (coded as 4)”. A higher coded value indicated a longer subjective distance from the epidemic.

### 2.4 Data analyses

First, missing data and attrition analyses were conducted to identify whether the data were missing completely at random and whether the 402 retained participants differed from the 124 respondents absent at Time 2. Second, statistical assumption testing, descriptive statistics, and correlation analyses were executed for the key variables using SPSS (version 26). Third, confirmatory factor analysis was performed for each latent construct with Mplus (Version 8.3). Fourth, a structural model was employed to test the research hypotheses. Fifth, the mediating effects were estimated using 1000 bias-corrected bootstraps. The weighted least squares with mean and variance adjusted (WLSMV) estimator was used for structural equation modeling. Structural equation modeling was used for two reasons. First, it includes the assessment of measurement errors to validate instruments. Second, it can test complex relationships among multiple predictors, mediators, and outcomes [36], allowing the specification and examination of our research model based on the PMT.

## 3. Results

### 3.1 Missing data and attrition analyses

In the analytic sample (*n* = 526), the missing values of the study variables ranged from 0% to 34.41%, totaling 11.84% missing data. Little’s Missing Completely at Random (MCAR) Test was performed, and our missing data showed a significant deviation from MCAR, χ^2^ (439) = 520.57, *p* = .004. For participant’s gender, age, education level, residence, health risk attitude, and health locus of control, no data were missing. For subjective distance from the epidemic, threat appraisal, and coping appraisal, 23.57% of values were missing due to participant attrition. For the items of protective behavior, 23.76% to 34.41% of values were missing due to participant attrition and item inapplicability. Full Information Maximum Likelihood (FIML), the default method to deal with missing data in Mplus (Version 8.3), was utilized to handle the missing data in the study sample.

Subsequently, *t*-tests and chi-square tests were executed to identify whether the 301 participants with no missing values differed from the 225 participants with missing values. Results showed that younger participants were more likely to have missing values, *t* (524) = 2.01, *p* = .045, but there were no significant differences on the other variables: for gender, χ^2^ (1) = 2.08, *p* = .150; for education level, *t* (524) = −0.49, *p* = .623; for residence, χ^2^ (2) = 4.03, *p* = .134; for health risk attitude, *t* (524) = 0.56, *p* = .576; for health locus of control, *t* (524) = 0.71, *p* = .475; for threat appraisal, *t* (400) = 1.88, *p* = .061; for coping appraisal, *t* (400) = −1.22, *p* = .225; for subjective distance from the epidemic, *t* (400) = −1.15, *p* = .251.

Also, *t*-tests and chi-square tests were executed to identify whether the 402 retained participants differed from the 124 respondents absent at Time 2. Results showed that no significant differences on the variables assessed at Time 1 were observed between the retained and non-retained participants: for gender, χ^2^ (1) = 0.27, *p* = .601; for age, *t* (524) = 1.37, *p* = .170; for education level, *t* (524) = 0.24, *p* = .807; for residence, χ^2^ (2) = 2.00, *p* = .369; for health risk attitude, *t* (524) = 0.72, *p* = .470; for health locus of control, *t* (524) = –1.03, *p* = .303. In sum, the sample did not experience differential attrition in terms of gender, age, residence, health risk attitude, and health locus of control.

### 3.2 Statistical assumption testing, descriptive statistics, and correlations

The statistical assumptions for structural equation modeling were evaluated, including normality, multivariate outliers, and multicollinearity. The normality of the observed variables was tested via skewness and kurtosis. Results showed that the skew values were between −1.806 and 2.664, and the kurtosis values were between −1.660 and 6.386. It has been argued that data are considered to be normal if the skewness is between −2 and +2 and the kurtosis is between −7 and +7 [37]. Thus, our data met the normality assumption, except for one item with a skew value (equal to 2.664) larger than 2. Mahalanobis distance showed there were three multivariate outliers, but none had Cook’s distance greater than 1, indicating they were not influencing the model and hence were retained [38]. Multicollinearity was tested by examining bivariate correlations, which showed that all the correlations (ranging from .001 to .63) were less than .85, indicating no severe multicollinearity.

The descriptive statistics and correlations for the key variables are presented in Table 1. It was found that participants’ health risk attitude was positively associated with health locus of control and protective behavior, but was not significantly associated with threat appraisal or coping appraisal. Health locus of control was positively associated with coping appraisal, but was not significantly associated with threat appraisal or protective behavior. Threat appraisal was negatively associated with coping appraisal, but was not significantly associated with protective behavior. Coping appraisal was positively associated with protective behavior.

**Table 1:** Descriptive statistics and correlations for the study variables.

Variable	*M ± SD*	1	2	3	4
1. Health risk attitude	3.93 ± 0.49				
2. Health locus of control	4.32 ± 0.62	.16***			
3. Threat appraisal	2.46 ± 0.73	–.04	–.01		
4. Coping appraisal	4.80 ± 1.11	.03	.11*	–.47***	
5. Protective behavior	3.93 ± 0.71	.16**	.04	.06	.14**

*Note*.

* *p* < .05

** *p* < .01

*** *p* < .001. For health risk attitude, scores ranged from 1 to 5, with higher means indicating higher health risk aversion. For health locus of control, scores ranged from 1 to 6, with higher means indicating more internal health locus of control. Threat appraisal was rated from 1 to 5, and higher means indicated participants appraised the virus more as a threat. Coping appraisal was rated from 1 to 7, and higher means indicated higher coping appraisal. For protective behavior, scores ranged from 1 to 5, and higher means indicated more frequent protective behaviors.

### 3.3 Structural model

A structural model was developed including health risk attitude and health locus of control as the predictors, threat appraisal and coping appraisal toward the COVID-19 pandemic as the mediators, and protective behavior as the outcome, along with gender, age, education level, residence, and subjective distance from the epidemic as the control variables. The results showed that the final model fit the data well, χ^2^ (458) = 688.06, *p* < .001, CFI = .950, TLI = .943, RMSEA = .031, SRMR = .084. The model fit was acceptable as the CFI and TLI values were larger than .90, and the RMSEA and SRMR values were smaller than .08 [39]. It should be noted that the chi-square value did not serve as a criterion for assessing the goodness of fit, due to the sample size [40].

**Fig 2:**
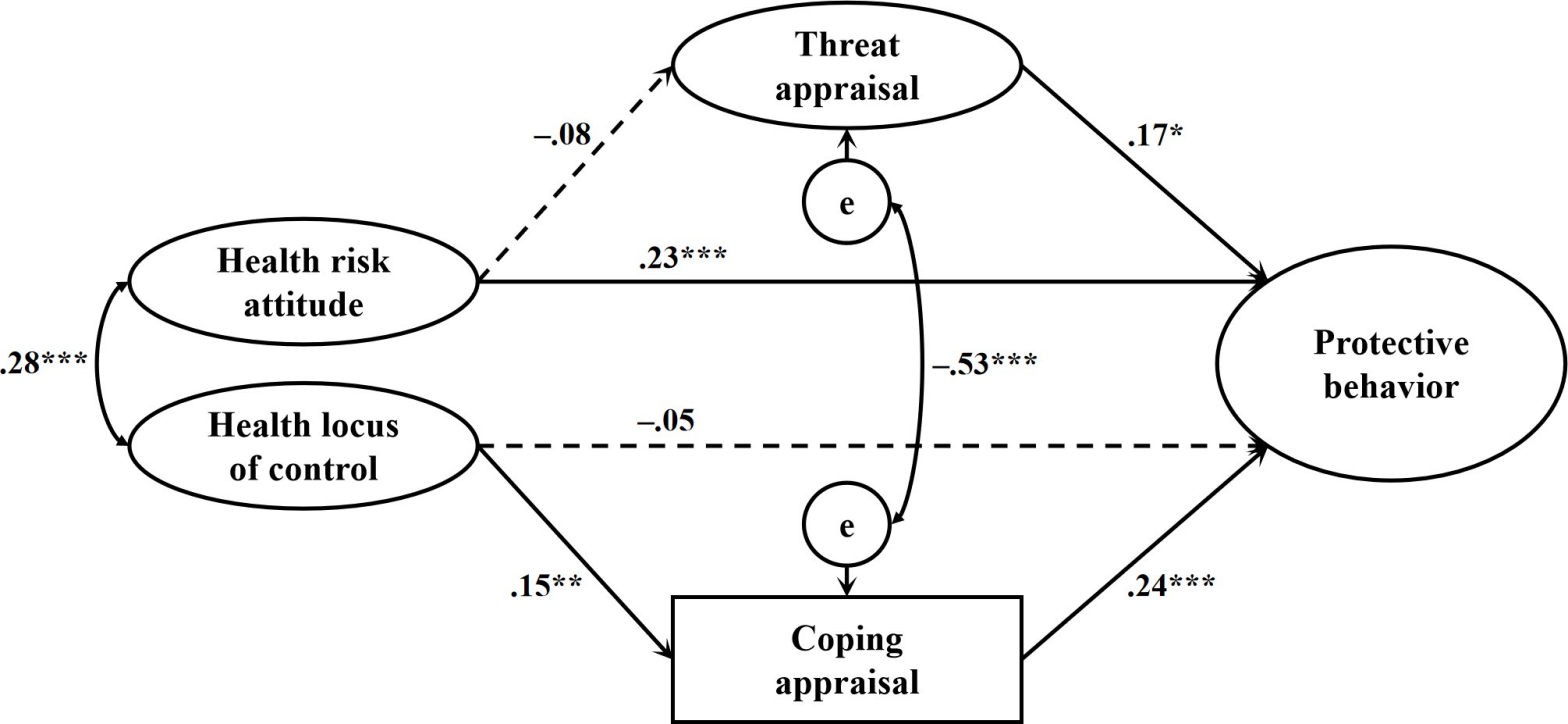
Effects of health-related dispositions on citizens’ appraisals toward the COVID-19 pandemic and protective behavior. *Note*. Values presented are standardized coefficients. χ^2^ (458) = 688.06, *p* < .001, CFI = .950, TLI = .943, RMSEA = .031, SRMR = .084. The dash lines represent non-significant paths. Control variables are not shown for parsimony. * *p* < .05, ** *p* < .01, *** *p* < .001.

As shown in Fig 2, the direct effect of health risk attitude on protective behavior was significant (β = .23, *p* < .001), whereas the direct effect of health locus of control on protective behavior was not significant (β = −.05, *p* = .444). Moreover, the total effect of health risk attitude on protective behavior (without the mediator) was significant (β = .22, *p* < .001), whereas the total effect of health locus of control on protective behavior was not significant (β = −.02, *p* = .771). These results indicated that Hypothesis 1a was supported whereas Hypothesis 1b was not. Health risk attitude was not significantly associated with threat appraisal (β = −.08, *p* = .151), which was positively associated with protective behavior (β = .17, *p* = .011). Health locus of control was positively associated with coping appraisal (β = .15, *p* = .007), which, in turn, was positively associated with protective behavior (β = .24, *p* < .001). Additionally, the indirect effect of health risk attitude on protective behavior via threat appraisal was not significant, β = −.013, 95% CI = [−.045, .006]. However, coping appraisal fully mediated the effect of health locus of control on protective behavior, as the indirect effect was significant, β = .036, 95% CI = [.001, .083]. These results indicated that Hypothesis 2a was not supported but Hypothesis 2b was supported.

For the control variables, age was positively correlated with protective behavior (β = .27, *p* < .001). Women reported higher levels of threat appraisal toward the COVID-19 pandemic than men (β = .17, *p* = .002). No other significant effects regarding the control variables were found. With regard to overall variance, 18.4% of the variance of protective behavior, 4.4% of the variance of threat appraisal, and 4.8% of the variance of coping appraisal were accounted for in the study sample.

## 4. Discussion

### 4.1 Effects of health risk attitude on citizens’ threat appraisal and protective behavior

According to the results, health risk attitude (from risk preference to risk aversion) had a direct and positive effect on citizens’ protective behavior, without an indirect effect mediated by threat appraisal toward the COVID-19 pandemic. The direct and positive effect is congruent with the prior studies suggesting a strong link between health risk aversion and self-preservation behavior [22,41]. Those citizens with higher levels of health risk aversion were inclined to take fewer health risks in daily life, and accordingly, were inclined to avoid health risks brought by inappropriate behaviors and to decrease health risks by performing protective behaviors during the COVID-19 pandemic. In contrast, those citizens with higher levels of health risk preference were less likely to put in place self-protection strategies toward possible negative health consequences evoked by the pandemic [42]. Moreover, consistent with previous research, threat appraisal was positively associated with protective behavior, which supports the PMT. The direct effect of health risk attitude on protective behavior without affecting threat appraisal implies the inertia of health risk attitude and rationality of threat appraisal. More specifically, health risk attitude would predict most protective behaviors automatically and effectively once the circumstances contain any health risk. However, threat appraisal is a comparatively rational process, which needs individuals to evaluate the external environment seriously, and thus is more context-specific. In conclusion, both health risk attitude, a relatively stable dispositional factor, and threat appraisal, a cognitive assessment of health threat in a specific situation, affected protective behavior.

Unexpectedly, this study did not find a significant effect of health risk attitude on threat appraisal toward the COVID-19 pandemic. Moreover, there was no significant correlation between the two. This may be due to the situational dependency of threat appraisal and the novelty of the COVID-19 pandemic. The pandemic was so novel that people even experts had no experience with it [43]. In this context, people’s threat appraisal might be more influenced by external environmental factors including media, real pandemic risk, and descriptive norms [44–48], rather than subjective health risk attitude, which is to some extent shaped by experience. Further, it is plausible to surmise that familiarity with certain health-related situations (e.g., the COVID-19 pandemic) may be more influential than individuals’ attitudes toward health risks when assessing health-threatening situations. Hence, health risk attitude failed to predict citizens’ threat appraisal toward the COVID-19 pandemic, and accordingly, no significant indirect effect of threat appraisal was found in the present study. These results combined with the significant effect of health risk attitude on protective behavior suggested that, there might be other mediators, or that health risk attitude affected protective behavior directly [49–51]. Nevertheless, further research is needed to determine the above possibilities.

### 4.2 Effects of health locus of control on citizens’ coping appraisal and protective behavior

The results of this study showed that health locus of control predicted protective behavior which was fully mediated by coping appraisal toward the COVID-19 pandemic. Those citizens with a strong internal health locus of control had a belief that they had control over the outcome of events concerning their health [52]. This belief promoted the citizens’ perceived response efficacy and perceived self-efficacy toward the health threats evoked by the pandemic [13]. In this way, the citizens with a strong internal health locus of control had higher levels of coping appraisal toward the COVID-19 pandemic, and then conducted more protective behaviors, which extends the PMT successfully. In contrast, those citizens with a weak internal health locus of control tended to believe that health related events were a result of external factors and were out of control, which might diminish their coping appraisal toward the COVID-19 pandemic and hence implemented less protective behaviors [53].

There were no significant direct and total effects of health locus of control on citizens’ protective behavior, which is congruent with a prior study on protective behavior against the COVID-19 pandemic which only found an indirect effect [29]. It might be due to that health locus of control is a very general and abstract factor that fundamentally explains an individual’s attribution tendency regarding self related matters [54], whereas combating the COVID-19 pandemic relied heavily on efforts of governments and other external forces beyond self [55]. This was particularly the case in China. Thus, health locus of control alone was not sufficient to directly influence citizens’ protective behavior in response to the pandemic. Yet such internal/external attribution tendency did affect individuals’ cognitive coping appraisal toward the pandemic, which in turn impacted their protective actions. In brief, the non-significant direct and total effects of health locus of control on citizens’ protective behavior underscore the mediating process through coping appraisal.

### 4.3 Limitations and future directions

Firstly, our sample was from Beijing, China, thus the findings could be culturally specific. It would be desirable to conduct such studies in more culturally diverse samples to examine the generalizability of our findings [56]. Secondly, this study relied on self-report Likert questionnaires, which might lead to common method biases. It would be beneficial to include other measurements in future research (e.g., a diary-based measure for protective behavior). Thirdly, it did not measure all the study variables at two time points, which might weaken the causality. Hence, a longitudinal design is preferred. Fourthly, rating of the health risk attitude items (from “not at all risky” to “extremely risky”) may cause some participants to evaluate the objective risky situation rather than subjective risk attitude. Future research would benefit from more appropriate approaches, such as rating the willingness or frequency to take health-related risky actions. Moreover, there might be a disconnection between the health risk of daily situations and the pandemic. Future research could add items involving risky situations in public health emergencies (e.g., “refusing to get a vaccine during a deadly pandemic”) to assess health risk attitude more comprehensively. Fifthly, the measure of coping appraisal included only two items, which may weaken its robustness. More reliable measurement is required. Sixthly, there might be other potential mediators, such as hygiene consciousness or psychological reactance toward protective behavior [29,57]. Further research is needed to identify these possible underlying mechanisms. Lastly, protective behavior could be impacted by other dispositional factors, such as cognitive reflection [58] and moral disengagement [59], which could be explored in future studies.

## 5. Conclusion

By implementing a survey including two waves of data, this study examined the effects of health risk attitude and health locus of control on threat and coping appraisals toward the COVID-19 pandemic and protective behavior of citizens, as well as the respective mediating effects of threat and coping appraisals toward the COVID-19 pandemic. The results showed that health risk attitude (from risk preference to risk aversion) had a direct and positive effect on citizens’ protective behavior, without an indirect effect mediated by threat appraisal toward the COVID-19 pandemic; internal health locus of control did not directly associate with citizens’ protective behavior, but had an indirect effect on it fully through coping appraisal toward the COVID-19 pandemic.

This study has both theoretical and practical implications. Theoretically, our findings suggest that health-related dispositional factors matter besides situation-based threat and coping appraisals, which is an extension of the PMT. Practically, the successful extension of health locus of control in the PMT suggests, it is important to develop more internal health locus of control so that people would have more positive coping appraisal and thus act more positively to confront the pandemic. Internal health locus of control could be developed effectively by daily public education on health knowledge and healthy lifestyle [60]. Regarding the maladaptive path in the PMT, results showed that threat appraisal was more dependent on context-specific information. Therefore, it is recommended to give full play to the guiding role of the government and the media to raise citizens’ awareness of the epidemic risks, such as by releasing more detailed and relevant information about the epidemic [61,62]. In addition, though this study did not extend the PMT with health risk attitude successfully, it could affect individuals’ protective behaviors directly. Therefore, guiding the public to shape cautious health risk attitude, such as launching comprehensive educational campaigns that explain health risks, is also necessary.

## Supporting information

S1 File Data files, syntax files, and log files for the analyses. (ZIP)

S1 Table Factor loadings for measurements of the study variables. (DOCX)
