# Supplementary material for: Effects of health-related dispositions on citizens’ appraisals toward the COVID-19 pandemic and protective behavior
Source: PLoS One. 2024 Sep 5;19(9):e0305995. doi: 10.1371/journal.pone.0305995 (PMC11376525; doi:10.1371/journal.pone.0305995)
Supplement: S1 File — (ZIP) [file pone.0305995.s001.zip › S1 Zip File/Explanatory Memo.docx]

**Explanatory Memo**

There are six files, including two data files, two syntax files, and two log files for the analysis.

**Data.sav**

This file includes the complete raw data collected in the study. The items are shown as labels in the data set. Regarding the variable names:

“HRA01_1~ HRA09_1” indicates the nine items of health risk attitude;

“HLC01_1~HLC05_1” indicates the five items of health locus of control;

“PB01_2~ PB08_2” indicates the eight items of protective behavior;

“TA01_2~ TA05_2” indicates the five items of threat appraisal toward the COVID-19 pandemic;

“CA01_2” and “CA02_2R (reverse coded)” indicate the two items of coping appraisal toward the COVID-19 pandemic;

“CAmean_2” indicates the mean of the two items of coping appraisal toward the COVID-19 pandemic;

“SubDis_2” indicates the subjective distance from the COVID-19 epidemic.

**Data for Mplus.dat**

This file is for structural equation modeling.

**Structural Model.inp**

This file is for structural equation modeling to test the theoretical model.

**Bootstrapping.inp**

This file is for bootstrapping to test the indirect effects.

**Structural Model.out**

This file is the output showing the results of the structural model.

**Bootstrapping.out**

This file is the output showing the results of the indirect effects.
