# Supplementary material for: Effects of health-related dispositions on citizens’ appraisals toward the COVID-19 pandemic and protective behavior
Source: PLoS One. 2024 Sep 5;19(9):e0305995. doi: 10.1371/journal.pone.0305995 (PMC11376525; doi:10.1371/journal.pone.0305995)
Supplement: S1 Table — (DOCX) [file pone.0305995.s002.docx]

**Table S1**

*Items and Factor Loadings of Health Risk Attitude, Health Locus of Control, Threat Appraisal and Protective Behavior*

| Items | Factor Loadings |
| --- | --- |
| **Health risk attitude** |  |
| Eating “expired” food products that still “look okay”. | .44 |
| Frequent binge drinking. | .62 |
| Ignoring some persistent physical pain by not going to the doctor. | .54 |
| Taking a medical drug that has a high likelihood of negative side effects. | .48 |
| Engaging in unprotected sex. | .60 |
| Never using sunscreen when you sunbathe. | .54 |
| Smoking a pack of cigarettes per day. | .66 |
| Buying an illegal drug for your own use. | .66 |
| Regularly eating high cholesterol foods. | .62 |
| **Health locus of control** |  |
| If I take care of myself, I can avoid illness. | .46 |
| Whenever I get sick it is because of something I’ve done or not done. | .57 |
| When I feel ill, I know it is because I have not been getting the proper exercise or eating right. | .58 |
| People’s ill health results from their own carelessness. | .64 |
| I am directly responsible for my health. | .52 |
| **Threat appraisal** |  |
| I think I will probably get infected with COVID-19. | .60 |
| I think the COVID-19 epidemic in China is serious. | .43 |
| I feel close to the COVID-19 pandemic. | .65 |
| I worry about getting infected with COVID-19. | .84 |
| I feel vulnerable to the COVID-19 infection. | .84 |
| **Protective Behavior** |  |
| In the past week, keep physical distance of at least one meter from others. | .72 |
| In the past week, wear a mask when there are other people around. | .49 |
| In the past week, avoid the spaces that are closed, crowded or involve close contact. | .75 |
| In the past week, keep windows open for ventilation when gathering indoors. | .66 |
| In the past week, thoroughly clean hands with either an alcohol-based hand rub or soap and water. | .55 |
| In the past week, avoid touching your eyes, nose, and mouth. | .71 |
| In the past week, clean and disinfect surfaces of objects, such as door handles and phone screens. | .58 |
| In the past week, when eating around a table with people, use communal serving chopsticks and spoons or implement individual serving of food. | .72 |
